# A capsid hinge region in European hepatitis E virus links mutations to genotype, virion surface properties, and environmental circulation

**DOI:** 10.1128/aem.01445-26

**Published:** 2026-08-28

**Authors:** Samy Kasmi, Guillaume Sautrey, Cédric Hartard, Fabienne Quilès, Jean-Luc Bailly, Alexis de Rougemont, Hélène Jeulin, Raphael E. Duval, Christophe Gantzer, Evelyne Schvoerer

**Affiliations:** 1 Laboratoire de Virologie, CHRU de Nancy Brabois 731944 , Vandœuvre-lès-Nancy, France; 2 Université de Lorraine, CNRS, LCPME 137665, Nancy, France; 3 Laboratoire Microorganismes: Génome et Environnement (LMGE), Université Clermont Auvergne 27006, Clermont-Ferrand, France; 4 Centre National de Référence des virus des gastro-entérites, Centre Hospitalier Universitaire Dijon Bourgogne 36659, Dijon, France; 5 UMR PAM A 02.102 Procédés Alimentaires et Microbiologiques, Université Bourgogne Europe/ INRAe/ L'Institut Agro Dijon, Dijon, France; 6 Laboratoire de Virologie Sérologie, Centre Hospitalier Universitaire Dijon Bourgogne, Dijon, France; 7 Université de Lorraine 137665, Nancy, France; University of Nebraska-Lincoln, Lincoln, Nebraska, USA

**Keywords:** hepatitis E virus, ORF2 capsid protein, environmental virology, mutation motifs, structural modeling, hydrophobicity, viral transmission

## Abstract

Hepatitis E virus (HEV) exhibits genetic diversity associated with distinct public health outcomes and circulation patterns across interconnected human and environmental reservoirs. Most studies have focused on genome-wide diversity, revealing relationships between genotype, virulence, and dissemination routes. However, the role of localized mutations in the capsid protein (ORF2), which may influence the extracellular fate and ecology of HEV through changes in surface biological and physicochemical properties of virions, received limited attention. Here, we combined large-scale analysis of European HEV sequences, structural modeling, and physicochemical characterization to investigate such variations in ORF2. Analysis of ORF2 sequences on a genomic segment highly reported in GenBank, derived from patients and the environment, revealed recurrent mutation motifs (single H354Y and triple H354Y–G357S–V364I motifs) located in a conserved capsid hinge region connecting the middle (M) and protruding (P) domains of the protein. These motifs displayed distinct frequency patterns according to sequence sources and were associated with genotypes 3 and 1, respectively. AlphaFold-based modeling identified this region as a flexible interface between the M and P domains and indicated that mutations reduce predicted alignment error of the P domain relative to the overall protein model, consistent with altered exposure at the virion surface. *In vitro* analyses of synthetic peptides encompassing this critical region revealed increased hydrophobicity and structural rearrangements, supporting local modulation of capsid surface properties. Together, these results identify a structurally and physicochemically sensitive hinge region in the HEV capsid, suggesting a link between ORF2 sequence variations, genotype, virion surface properties, and environmental circulation.

**IMPORTANCE:**

Hepatitis E virus (HEV) is a major cause of viral hepatitis worldwide, which is transmitted through complex, genotype-linked dissemination routes between humans, animals, and environmental waters. Understanding the circulation dynamics of HEV virions is essential for improving environmental surveillance, risk assessment, and public health strategies within a One Health approach. By combining genetic, structural, and physicochemical analyses, this study highlights specific mutation motifs in a hinge region of the ORF2 capsid protein likely to link distinct distribution patterns of HEV across environmental reservoirs to potential changes in virion surface properties. Such mutation motifs may serve as molecular signatures for exploring virus persistence and dissemination in environmental contexts. Overall, this work provides a new framework for connecting viral genetic variation to environmental behavior beyond traditional genotype classification.

## INTRODUCTION

Hepatitis E virus (HEV) is a major cause of acute or chronic viral hepatitis worldwide, with an estimated global seroprevalence of 12.5% and approximately 20 million people newly infected per year (1, 2). The virus exhibits substantial genetic diversity across eight distinct genotypes (HEV-1 to HEV-8) circulating in humans, domestic/wild animal populations, and environmental water reservoirs, of which HEV-1 to HEV-4 are the main genotypes responsible for human infections (3, 4). Notably, HEV-1 and -2 are present in sub-Saharan Africa and East and South Asia as anthroponotic genotypes transmitted by the fecal-oral route via contaminated water, typically causing symptomatic infections in young adults and pregnant women (5). In contrast, HEV-3 and -4 are highly diverse, zoonotic viruses mainly isolated in humans and animals in industrialized countries, although water-linked transmission events, usually associated with HEV-1 and -2, cannot be excluded (6), and are particularly problematic in immunocompromised patients (5). Most studies in this field have primarily focused on genome-wide sequence variation of HEV, enabling the identification of genotype-specific patterns associated with virulence, host range, and dissemination routes (2, 7). In Europe, for example, where HEV-3 is predominant, the presence of the virus is heterogeneous, with an increasing number of autochthonous HEV cases observed for several years and marked regional HotSpots in seroprevalence (8–14). This diversity reflects the ability of HEV to circulate across diverse biological and environmental contexts, justifying the need for effective surveillance of HEV transmission, notably in industrialized countries with sanitation and safe water supplies (6, 15). However, exclusive genotyping-based approaches may overlook the contribution of localized mutations to viral behavior, especially when these mutations affect structural and physicochemical properties of virions (16). A deeper understanding of HEV transmission routes now requires integrating phylogenetic analyses with functional characterization of viral strains (17, 18). Indeed, beyond genetic classification, the ability of HEV virions to persist and circulate may be influenced by surface-exposed properties affecting specific virus-host interactions (e.g., receptor-mediated attachment at the cell surface) (19–21). These surface properties are also expected to govern non-specific, physicochemical interactions experienced by virions before encountering host cells. In particular, surface physicochemical properties, especially electrostatic charge and hydrophobicity, may influence various adsorption processes *ex vivo* (e.g., virion interactions with suspended organic matter and minerals in environmental matrices) (22, 23) and *in vivo* (e.g., virion interactions with biological macromolecules and extracellular polymers) (24), thereby affecting virus transport, persistence, and availability for subsequent infection. Understanding how localized capsid mutations modify these properties may therefore provide valuable insights into HEV environmental circulation and transmission.

HEV virions are 32–34 nm, non-enveloped, icosahedral particles enclosing a single-stranded RNA genome consisting of approximately 7,200 nucleotides (25). The capsid protein (ORF2) forms the outer architecture of virions, thereby mediating their surface physicochemical and biological properties. ORF2 is structurally organized into distinct protein domains, including the shell domain (S, buried in the capsid structure and facing inward), middle domain (M, at the inner/outer interface and partially exposed outward), and protruding domain (P, rising to the outer surface of the capsid) (26, 27). Several studies have reported genotype-associated mutations within the ORF2 amino acid sequence, often located in the P domain, displaying a higher degree of sequence variability compared to the S and M domains (21, 28, 29). This organization is consistent with the functional role of the P domain, which plays a key role in host interaction and immune recognition (16, 20, 25). Many of these substitutions have been associated with modulation of assembly, antigenicity, or infectivity (20, 21, 29); however, their broader physicochemical implications, notably regarding virion surface properties, remain largely unexplored. Moreover, besides the P domain, flexible or hinge-like ORF2 regions connecting protein domains may also represent HotSpots for variations affecting capsid structural dynamics and surface exposure (25, 27). In particular, recurrent mutation motifs in the M domain, i.e., H354Y, G357S, and V364I, have been identified in multiple sequence data sets in association with other substitutions (29), and are situated within a genome region contributing to phylogenetic signal (3, 4, 16), but their potential functional significance—especially given their location at the interface between the M and P domains (26)—remains unclear. These observations prompted us to specifically investigate whether mutations located in this putative hinge region could influence virion surface properties.

We therefore hypothesized that localized mutations within the M domain, as structurally sensitive regions of ORF2 likely to influence the relative organization or exposure of the P domain, could influence HEV environmental behavior by modulating virion surface properties. To test this hypothesis, we combined large-scale analysis of European HEV ORF2 sequences, including water-linked isolates, AlphaFold-based structural modeling of the protein, and physicochemical characterization of model peptides representing the putative hinge region of the M domain to investigate the mutations H354Y, G357S, and V364I. By these integrated approaches, our work aims to provide new insights into the role of capsid-level variation in HEV behavior and to complement genotype-based surveillance for a comprehensive understanding of HEV circulation in environmental and host-associated contexts.

## RESULTS

### Compiling human-derived, animal-derived, and “water-linked” ORF2 sequence libraries from European databases

**Fig 1:**
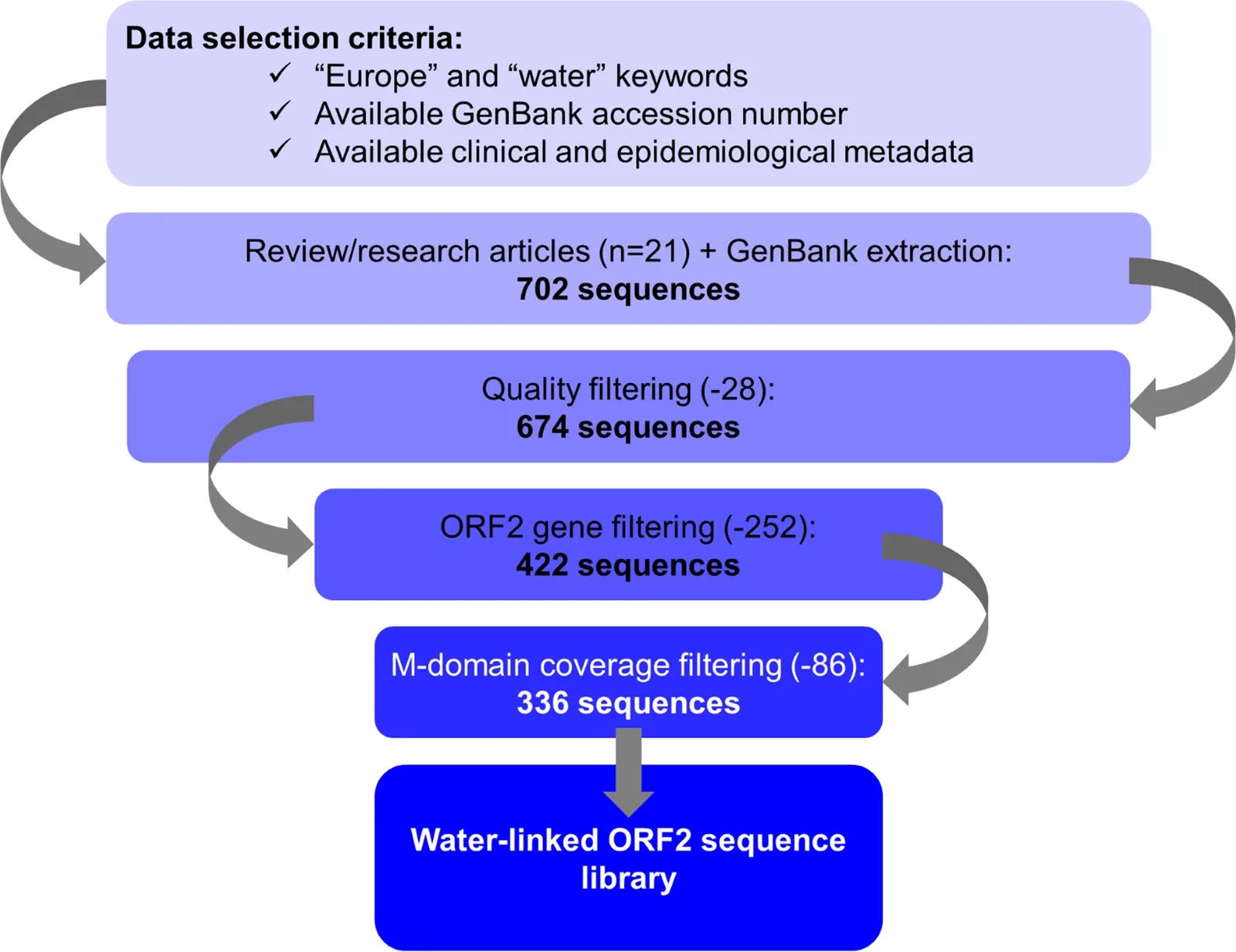
Flowchart illustrating the successive filtering steps leading from the initial set of water-linked HEV sequences detailed in Table 1 (*n* = 702) to the final water-linked ORF2 sequence library covering the ORF2 M domain (*n* = 336). The literature extraction was conducted via PubMed to identify articles based on the following criteria: available GenBank accession numbers, viral strains from Europe and potential water sources, and clinical/epidemiological information related to HEV.

A total of 2,641 human-derived and 1,184 animal-derived ORF2 sequences covering the ORF2 M domain of HEV isolates, accompanied by corresponding GenBank accession numbers, were extracted from European public databases for compiling human-derived and animal-derived ORF2 sequence libraries (both available in Tables S1 and S2). Additionally, 702 HEV GenBank sequences accompanied by detailed clinical and epidemiological information were pooled from 16 research articles mentioned in a recent meta-analysis devoted to assessing the prevalence of HEV in environmental water matrices (6), together with five further articles we found in the literature on the same topic (13, 30–49). The majority of these articles discussed complex HEV spread networks via wastewater and/or surface waters (rivers and lakes), without excluding combined water/human/animal transmission routes. Table 1 provides an overview of the main characteristics of these additional HEV sequences, including the number and type of sequences, their sources, and the potential modes of virus transmission. All the sequences were originally genotyped by the authors at the time of submission to GenBank, and the vast majority belonged to genotype 3 (HEV-3). As shown in Fig. 1 and detailed in Materials and Methods, a refinement of these 702 sequences was performed to focus on the ORF2 gene and cover the M domain specifically for the present study. This led to a third ORF2 sequence library (*n* = 336) we collectively designated as “water-linked” (available in Table S3).

**TABLE 1:** Distribution of water-linked HEV sequences (*n* = 702) according to virological/epidemiological features^*a*^

Country	Geographical region	No. of sequences	Source of sequences	Genotype	Subtype	Gene	Presumed HEV transmissions	Ref
France	Auvergne-Rhône-Alpes	15	WW, H, A	3	3f	ORF2	Environment (WW)	(30)
France	Auvergne-Rhône-Alpes	22	WW, H, A	3	3f, 3c, 3a	(n)FLG, ORF1	Environment (WW)	(31)
Germany	Berlin, Munich	41	WW, H, A	3	3f, 3c, 3a	ORF2, (n)FLG	Environment (WW, SW)	(32)
Italy	Central Italy	32	H, A	3	3f, 3c, 3a, 3e	ORF2, (n)FLG	Foodborne^*b*^	(33)
Italy	North East	174	H, A	3	3f, 3c, 3a, 3e	ORF2, ORF1, (n)FLG	Environment (WW, SW), foodborne	(34)
Italy	Central Italy	1	WW	4	4d	(n)FLG	Environment (WW)	(35)
Italy	Central Italy	94	WW, H, A	3, 1	3f	ORF1	Environment (WW, SW)	(36, 37)
Israel	North West	15	WW, SW, H, A	3, 1	3f, 3e	ORF1, (n)FLG	Environment (WW)	(38)
Italy	South Italy	13	WW, H	3, 1	N.I.	ORF1	Environment (WW)	(39)
Italy	North and South Italy	18	WW	3, 1	N.I.	ORF1	Environment (WW)	(40)
Netherlands	N.I.	63	WW, H, A	3	3f, 3c, 3a, 3e	ORF2	Environment (WW, SW), foodborne	(41)
Portugal	North and Central	2	WW	3	3i, 3f	ORF1	Environment (WW)	(42)
Slovenia	N.I.	30	A	3, 1	3e	ORF1, ORF2	Environment (WW, SW), zoonotic (pig)	(43)
Spain	Barcelona	24	WW, H, A	3	N.I.	ORF2, (n)FLG	Environment (WW, SW), zoonotic (pig)	(44)
Spain	Barcelona	1	WW	N.I.	N.I.	(n)FLG	Environment (WW)	(45)
Spain	Barcelona	5	WW, H	3	N.I.	ORF1, ORF2	Environment (WW, SW), zoonotic (pig)	(46)
Spain	N.I.	7	WW, H	3	3f	ORF2	Environment (WW)	(47)
Sweden	Gothenburg	7	WW	3	3c, 3i	ORF1	Environment (WW, SW), zoonotic (rodent)	(48)
Scotland	Edinburgh	90	WW, H	3	3f, 3c, 3a, 3e	ORF2	Environment (WW)	(49)
France	North-East	48	WW, H, A	3	3f, 3c, 3a	overlap, ORF2/ORF3	Environment (WW), zoonotic (rodent)	(13)
Spain	Barcelona	1	WW	N.I.	N.I.	(n)FLG	Environment (WW)	(45)

^*a*^ N.I., not indicated; WW, wastewater; H, human; A, animal; SW, surface water; (n)FLG, (near)full-length genome.

^*b*^ Rather “foodborne”—reference pathway for HEV in Europe.

Moreover, seven sequences associated with French HEV strains were added from our local collection. The HEV isolates come from six patients and one wild boar, and are contemporary with the public data mentioned above (6).

### Analyzing variation motifs in ORF2 at the positions 354, 357, and 364 in the European context

**Fig 2:**
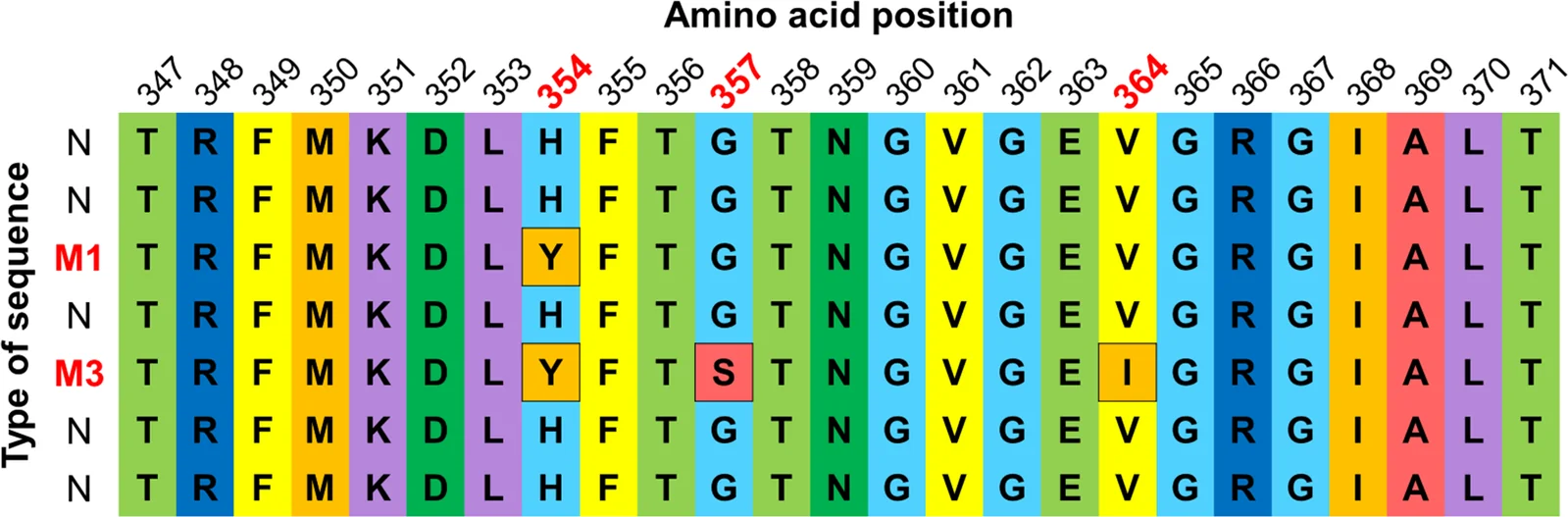
HEV ORF2 amino acid alignment in MEGA-12. Representative snapshot focusing on the mutation-prone positions (354, 357, and 364) in a conserved region of the M domain of ORF2, showing single H354Y (M1) and triple H354Y–G357S–V364I (M3) mutation motifs within native sequences (N).

As shown in Fig. 2, MEGA-12 software was used for highlighting single H354Y (M1) and triple H354Y–G357S–V364I (M3) mutation motifs within the aligned sequence data sets from the human-derived, animal-derived, and water-linked ORF2 libraries. No other motifs involving the residues 354, 357, and/or 364 were detected. Table 2 reports the detection frequency of M1 and M3 motifs as a function of the ORF2 library, whereas neither was detected within our French local collection. Analysis of the 2,641 human-derived ORF2 sequences identified 49 M1 motifs (1.86%) and 55 M3 motifs (2.08%), with a total of 3.94% detection for M1 or M3 together. In contrast, 10 M1 motifs (0.84%) and 1 M3 motif (0.08%) were detected within the 1,184 ORF2 sequences from the animal-derived library, with a total of 0.92% for M1 or M3 together. An intermediary pattern was observed within the 336 water-linked ORF2 sequences, with 1 M1 motif (0.30%) and 3 M3 motifs (0.89%), for a M1 + M3 total of 1.19%. From the three libraries together, the sequences harboring the M1 motif (3.00% total detection) or the M3 motif (3.05% total detection) were analyzed with the online genotyping tool provided by the National Institute for Public Health and the Environment of Netherlands (RIVM, https://mpf.rivm.nl/mpf/typingtool/hev/). Regardless of their origin, we classified 100% of M1-harboring sequences as belonging to genotype 3 according to the current RIVM genotyping tool, while 100% of M3-harboring sequences were classified as genotype 1. However, we compared these results with the clinical/epidemiological data available for the sequences within the water-linked library, and we observed that (i) 2/3 of the M3-displaying sequences, isolated from HEV patients in the United Kingdom, have been originally classified as genotype 3 (49); (ii) the M1-displaying sequence, from the same authors but isolated from wastewater samples, has been also originally classified as genotype 3; and (iii) the third M3-displaying sequence, isolated from Spanish wastewater samples, has been reported as unclassifiable (44). These discrepancies in genotyping support the putative hinge character of the residues 354, 357, and 364.

**TABLE 2:** Detection frequency of the M1 (H354Y) and M3 (H354Y–G357S–V364I) motifs within the human-derived, animal-derived, and water-linked ORF2 sequence libraries

Motif	Human-derived (*n* = 2,641)	Animal-derived (*n* = 1,184)	Water-linked (*n* = 336)	Total (*n* = 4,161)
M1	1.86%	0.84%	0.30%	3.00%
M3	2.08%	0.08%	0.89%	3.05%
Total (M1 + M3)	3.94%	0.92%	1.19%	

### AlphaFold modeling of the ORF2 capsid protein

**Fig 3:**
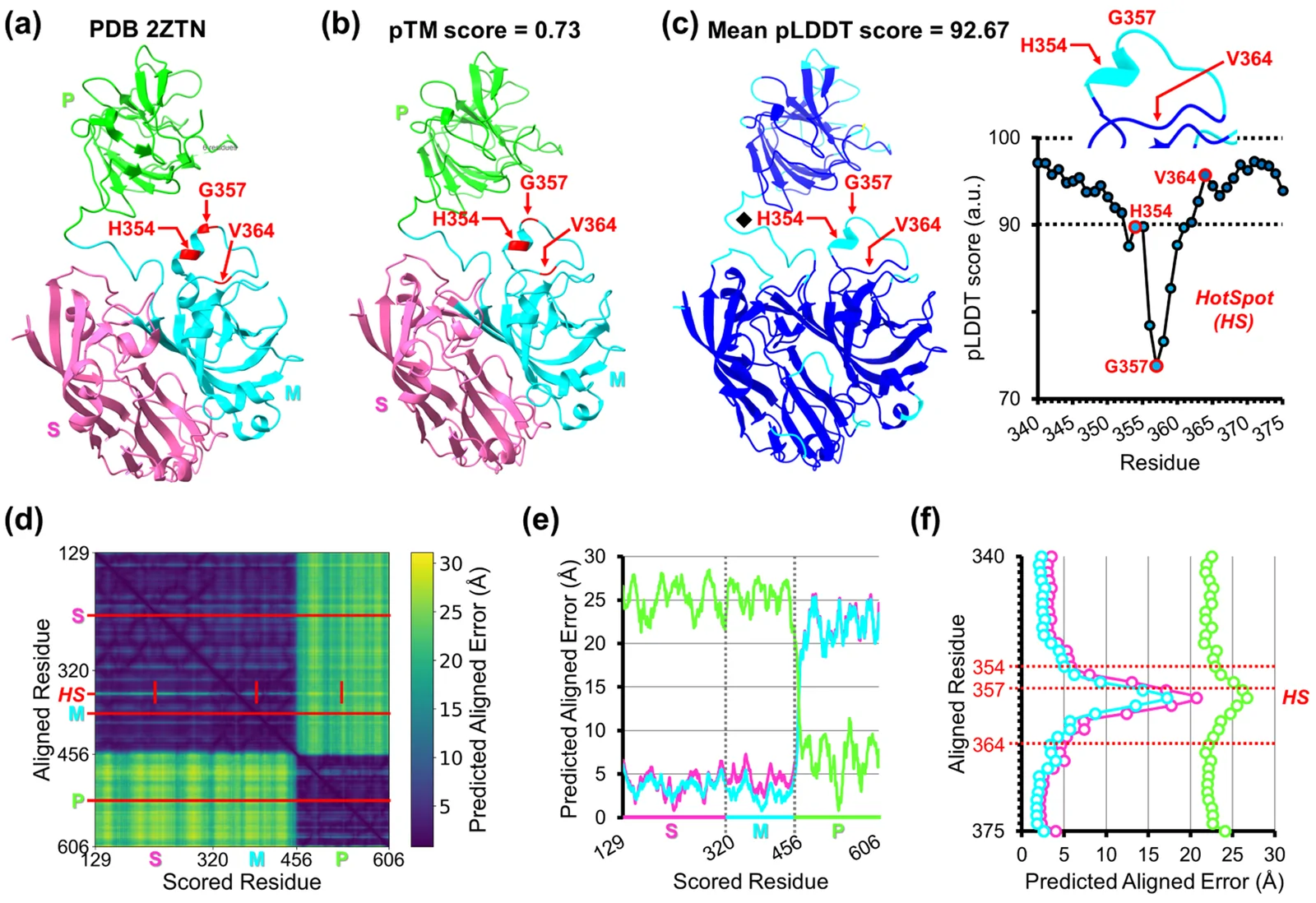
Structural analysis of the native, mutation-free, ORF2 capsid protein. The mutation-prone HotSpot (*HS*) is indicated by red. (**a**) Crystal structure (PDB id 2ZTN) (26) colored by protein domains. Pink: S domain (residues 129–319), cyan: M domain (320–455), and green: P domain (456–606). (**b**) Predicted structure and pTM score by AF3, colored as in panel a. (**c**) The same predicted structure but AF3-like colored by pLDDT score range. Dark blue: pLDDT > 90, light blue: 90 > pLDDT > 70, and yellow: 70 > pLDDT > 50. The graph shows the pLDDT score per residue in the *HS* region. (**d**) PAE matrix 3D plot of the predicted structure shown in panels b and c. S, M, and P domains, and boundary residues are indicated on x- and y-axes. The horizontal and vertical red lines correspond to the PAE 2D plots given in panels e and f. (**e**) PAE (y-axis) of three residues located in the middle of each protein domain (pink: M224, S domain; cyan: T338, M domain; and green: Q531, P domain) with respect to all protein residues (x-axis, graduated and colored by protein domains), which corresponds to the horizontal red lines in panel d. (**f**) Aligned residues (red) in the *HS* region (y-axis) as a function of their PAE (x-axis) with respect to the three residues representative of protein domains (pink: M224, S domain; cyan: T338, M domain; and green: Q531, P domain), which corresponds to the vertical red lines in panel d.

**Fig 4:**
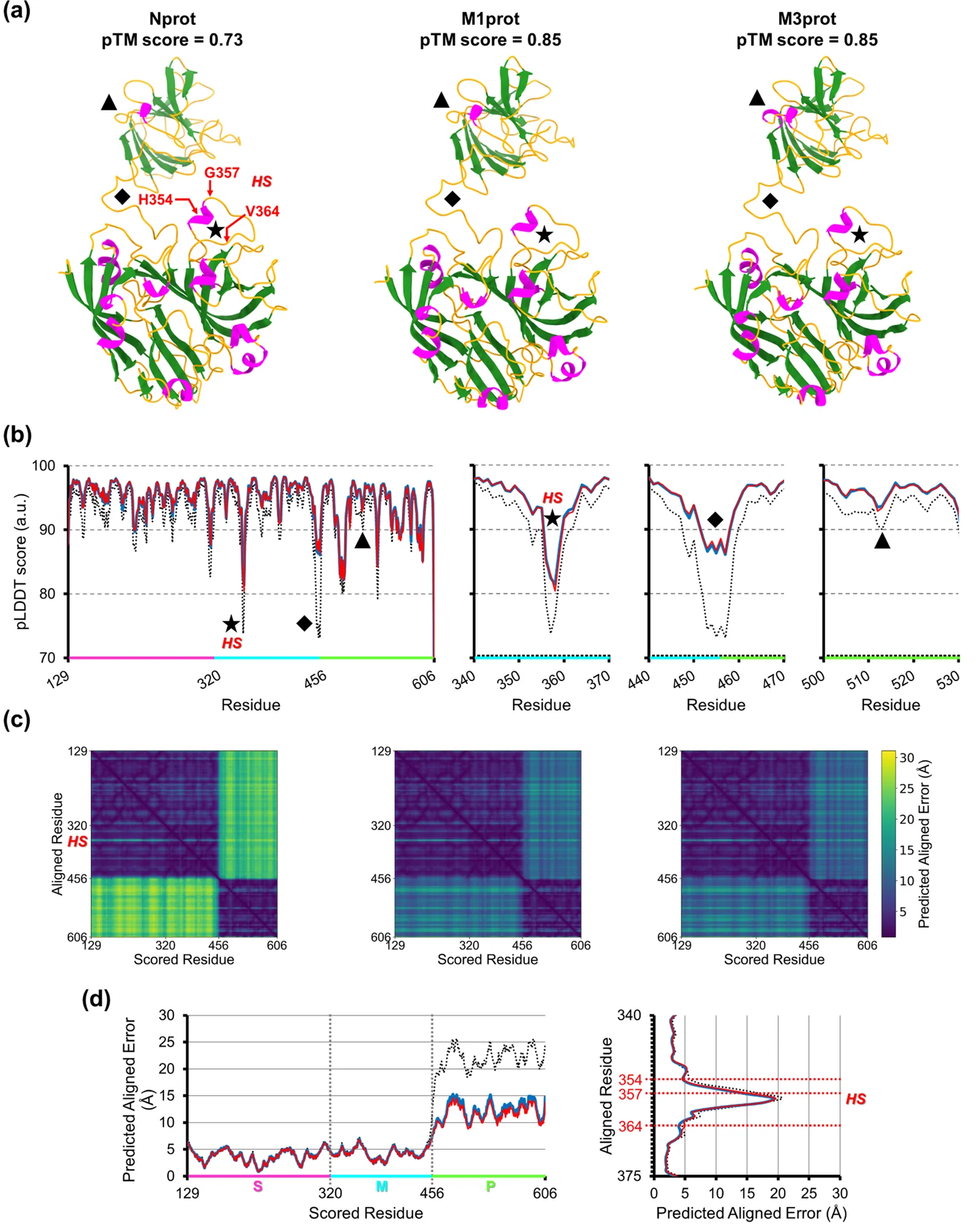
Comparison of AF3 predictions of native and mutant forms of the ORF2 capsid protein. The mutation-prone HotSpot (*HS*) is indicated in red. (**a**) AF3 predicted structures and pTM scores of the native form (Nprot), the single (H354Y), and the triple (H354Y, G357S, and V364I) mutant forms (M1prot and M3prot, respectively), colored by secondary structures (pink: α-helix, green: β-sheet, and orange: coil). (**b**) Plots of the pLDDT score per residue as a function of residue position for Nprot (dotted black line), M1prot (blue line), and M3prot (red line). The x-axes indicate protein domains using a color code as in Fig. 3 (pink: S domain 129–319, cyan: M domain 320–455, and green: P domain 456–606). Left panel: whole sequence. Right panel: focus on specific regions indicated by triangle, diamond, and star symbols. (**c**) PAE matrix 3D plots of the predicted structure shown in panel a. From left to right: Nprot, M1prot, and M3prot. (**d**) Left panel: PAE of the M224 residue (middle of S domain) with respect to all protein residues in Nprot (dotted black line), M1prot (blue line), and M3prot (red line). The x-axis is graduated and colored by protein domains as in panel b. Right panel: Aligned residues (red) in the *HS* region (y-axis) as a function of their PAE (x-axis) with respect to the M224 residue (middle of the S domain) in Nprot (dotted black line), M1prot (blue line), and M3prot (red line).

According to published X-ray crystallographic data (26), the three mutation-prone residues H354, G357, and V364 are located in a surface-exposed HotSpot (*HS*) of the M domain spatially close to the P domain of the HEV capsid protein (Fig. 3a). To assess the potential consequences of the amino acid changes at these sites on the structure and properties of the ORF2 capsid protein, native and mutant molecular models were predicted with AF3 (50) (Fig. 3b through f and Fig. 4, respectively). The native model from AF3 (Fig. 3b and c) offered satisfactory confidence metrics (pTM score = 0.73 and mean pLDDT score = 92.67) and fits well visually with the crystal structure 2ZTN from PDB shown in Fig. 3a, while some low-confidence regions were locally observed (Fig. 3c through f). On the one hand, the pLDDT per residue indicates a sharp valley just in the *HS* region (residues 353–361) with a minimum (pLDDT = 73.82) located on the mutant-prone G357 residue (Fig. 3c, right panel). On the other hand, the predicted aligned error (PAE) plot (Fig. 3d) shows high PAE between 129–456 and 456–606 residue groups (mean PAE ≈ 20–25 Å) (Fig. 3e). Additionally, a thin PAE increase by 20–25 Å was observed just in the *HS* region (Fig. 3d), and with regard to all domains with a misalignment centered on the residue T358 (Fig. 3f).

The second step was to compare, in Fig. 4, the AF3-predicted structures of the ORF2 protein in native form (Nprot) with the single H354Y and the triple H354Y–G357S–V364I mutant forms (M1prot and M3prot, respectively). As shown in Fig. 4a, only discrete impacts of mutations were observed in the predicted molecular models of M1prot and M3prot compared to Nprot, except for a slight expansion of the unique α-helix in the P domain (triangle symbol) of M3prot (residues 511–518) in comparison with Nprot (residues 516–518). However, all mutation motifs significantly impacted the confidence metrics, first by an increase of pTM scores from 0.73 for Nprot to 0.84–0.85 for M1prot and M3prot. The pLDDT scores also improved through mutations (Fig. 4b), shifting from a mean score 92.67 ± 4.40 for Nprot to 94.80 ± 3.48 and 94.77 ± 3.52 for M1prot and M3prot, respectively. Whatever the mutation motif involved, the pLDDT enhancement is particularly sharp in critical areas such as the *HS* and the M-to-P linker regions (compare symbols between Fig. 4a and b), by shifting from around 73–74 to 85–88. A strong impact of mutations was likewise captured in the PAE matrix 3D plots (Fig. 4c). The high misalignment of the P domain with respect to both S and M domains by 20–25 Å in Nprot was less pronounced in mutant forms, decreasing to 10–15 Å, regardless of the mutation motif (Fig. 4d, left panel), while the misalignment of the *HS* region noticed in the Nprot prediction was unchanged in the mutant forms (Fig. 4d, right panel).

### Structural impact of mutations in model peptides

**Fig 5:**
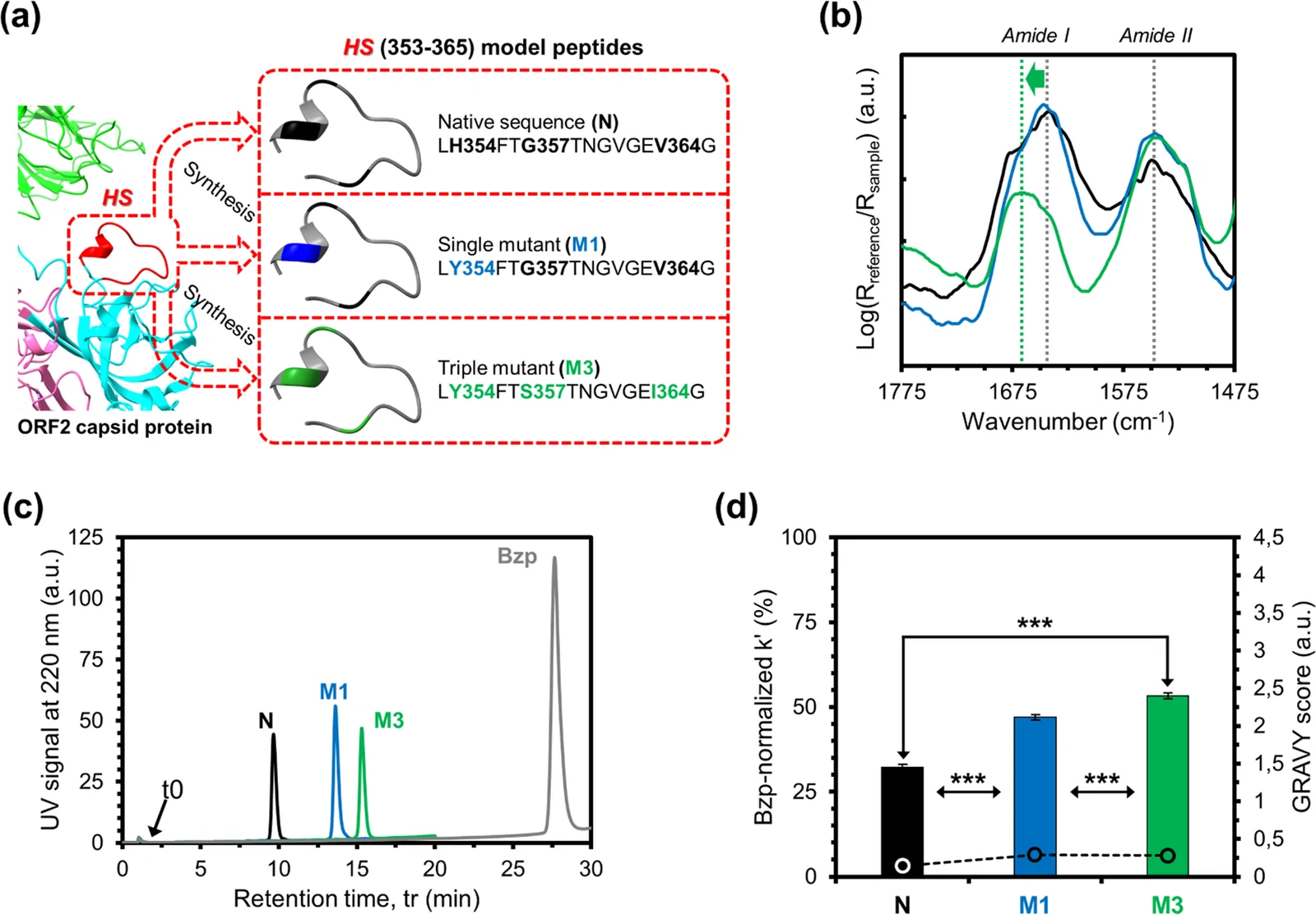
Structural and physicochemical investigation of model peptides. (**a**) Selection of model peptides derived from the *HS* region (red) in the ORF2 capsid protein (residues 353–365). The sequence of peptides displays no mutation (N, black), one mutation (M1, blue), or three mutations (M3, green). (**b**) IR-ATR spectra (Amide I and II regions) of 1 mg/mL aqueous solutions of N (black), M1 (blue), and M3 (green) in micellar pH 7 buffer. (**c**) Compilation of representative chromatograms of benzophenone (Bzp), N, M1, and M3 with UV detection. An injection-induced perturbation is visible as a t0 marker. (**d**) Plot of Bzp-normalized *k*′ (bars, left y-axis, *** means *P*-values <0.001) and GRAVY scores (dashed line, right y-axis) of N, M1, and M3. Both y-axes are graduated on the entire rising hydrophobicity scale (0%–100% for Bzp-normalized *k*′ and 0–4.5 for GRAVY).

The possible structural and physicochemical impacts of H354Y, G357S, and V364I mutations on the *HS* region of the ORF2 capsid protein were explored by means of three synthetic model peptides consisting of a 13-residue sequence corresponding to the positions 353–365 of the whole protein (Fig. 5a). Infrared spectroscopy in attenuated total reflectance (IR-ATR) was used for detecting potential changes in the secondary structure of model peptides in aqueous solution. Figure 5b shows the IR-ATR spectra of N, M1, and M3 model peptides in the Amide I and II absorption band region, emanating from the peptide bonds and widely used in probing secondary structures (e.g., α-helix, β-turns, or β-sheets) in amino acid polymers (51). Amide I bands absorbed at 1,678, 1,644, and 1,630 cm^−1^ for N and M1 peptides (detected through second-derivative spectra, data not shown). The profile of Amide I bands from the M3 peptide was shifted with respect to those from N and M1, with bands at 1,677, 1,660, and 1,639 cm^−1^. They indicated a slightly higher content of β-sheets and/or β-turns in the M3 peptide due to the triple mutation H354Y–G357S–V364I, while the single H354Y motif has almost no effect on the secondary structure of the M1 peptide.

### Hydrophobicity change in model peptides

Considering the physical chemistry, especially hydrophobicity, the grand average of hydropathy (GRAVY) of model peptides was calculated according to the Kyte and Doolittle scale that attributes a hydropathy index (HI) for each residue from −4.5 (polar) to +4.5 (apolar) (52). The GRAVY is the sum of HI of all residues divided by the sequence length of the peptide, resulting in a weighted score between −4.5 (hydrophilic) and +4.5 (hydrophobic). The single mutation H354Y from the polar histidine (−3.2 HI) to the moderately polar tyrosine (−1.3 HI) should increase the hydrophobicity of the M1 peptide in comparison with the N peptide, as shown by the corresponding GRAVY scores +0.14 (N) and +0.29 (M1). However, the hydrophobicity of the triply mutated M3 peptide should not significantly differ from M1, returning the GRAVY score +0.28 for M3. The relative hydrophobicity of model peptides was evaluated *in vitro* through reverse-phase high-performance liquid chromatography (RP-HPLC), where the retention time (tr) depends on the hydrophobicity of solutes: the more a solute is hydrophobic, the more tr rises, while all hydrophilic solutes have the same, minimal, retention time (t0) whatever their hydrophilic degree (53). This allowed focusing on the hydrophobic degree and calibrating it against a highly hydrophobic benchmark such as benzophenone (Bzp) as a normalizer (Fig. 5c and d). The Bzp-normalized retention factor *k*′ of peptides was calculated and plotted in Fig. 5d to provide a quantitative experimental scale of hydrophobicity rising from 0% (low hydrophobic) to 100% (highly hydrophobic). N, M1, and M3 peptides previously assessed as barely hydrophobic from GRAVY scoring (+0.14, +0.29, and +0.28, respectively, which can be seen on a 0/+4.5 scale since negative scores are related to hydrophilicity) appeared hydrophobic as well (Fig. 5d). On this scale, the native peptide N is moderately hydrophobic (*k*′ was 32.3% ± 0.2%). Interestingly, the M1 peptide (*k*′: 47.0% ± 0.2%) is significantly more hydrophobic than the N peptide, as concluded from GRAVY scoring, but M3 (*k*′: 53.3% ± 0.2%) is also significantly more than M1, despite no real difference detected from corresponding GRAVY scores.

## DISCUSSION

Taken together, our results identify a hinge region within the ORF2 capsid protein of HEV (encompassing the residues 354, 357, and 364) as a key site that may influence HEV circulation across environmental and host reservoirs by modulating virion surface properties. This finding emerges from the convergence of analyses of ORF2 sequences extracted from public databases, combined with meta-analyses and cross-referencing with studies supporting water-linked viral transmission (Fig. 1; Table 1), structural modeling (Fig. 3 and 4), and physicochemical characterizations (Fig. 5).

First, although the M1 (H354Y) and M3 (H354Y–G357S–V364I) mutation motifs (Fig. 2) occur at low frequencies, they were recurrently detected across independent human-derived, animal-derived, and water-linked ORF2 sequence libraries, where both motifs displayed subtle but distinct distribution patterns (Table 2). Together, these observations support a combined viral-, host-, and environment-dependent interpretation. An additional layer of complexity arises from the association between mutation motifs and genotype background. In the present study, the M3 motif was exclusively observed in HEV-1 sequences, whereas the M1 motif was restricted to HEV-3 sequences. Indeed, the ORF2 residues Y354, S357, and I364 (i.e., the M3 motif) have been previously related to HEV-1, while the residues H354, G357, and V364 (i.e., the N motif) are linked to genotype 3 (29). We additionally observed single variations H354Y (M1 motif) within sequences we classified as genotype 3, suggesting that these mutations may occur independently of strict genotype-specific signatures. This raises the possibility that the distinct occurrence pattern of M1 and M3 motifs observed within the water-linked ORF2 sequence library, relative to the human- and animal-derived data sets, may reflect ecological processes associated with HEV circulation rather than genotype background alone. However, the specific mechanisms that could underlie this hypothesis, including any influence of environmental compartments, remain to be determined. While the segregation of M1 and M3 motifs may partly reflect phylogenetic structure, it also suggests that genotype-specific constraints could influence the accessibility or functional impact of mutations within this capsid region. Considering the ecological and epidemiological characteristics of HEV-1 and HEV-3, this observation raises the possibility that capsid-level variations affecting virion properties may be modulated by both environmental context and genetic background. Interestingly, one of the wastewater samples analyzed by Smith et al., as reported in the present study, harbored the M1 motif and was considered a genotype 3 (49), potentially representing a variant with altered environmental dissemination features. This will have to be further re-examined along with other potential HotSpots in capsid proteins. The pooling of HEV sequences would warrant regular studies targeting key genomic segments from various genotypes, and perhaps even HEV subtypes. Extending the present approach to larger international data sets, including HEV-1 and HEV-2 sequences from endemic regions outside Europe, would further help to determine whether the M1 and M3 motifs reflect broader genotype-associated ORF2 signatures or region-specific evolutionary and epidemiological contexts. In this field, a recent study exploring the transmission dynamics of HEV-3 subtypes across humans, animals, and environmental waters in Sweden reported a predominance of HEV-3f in animal reservoirs and HEV-3c in environmental waters, while both subtypes were detected in clinical isolates (54). However, since the data sets investigated cover different sampling periods spanning 12 years, this contrasting subtype distribution may also partly reflect temporal changes in HEV circulation, which should be considered when interpreting these findings. Despite this, the Swedish study provides complementary observations to our work while raising questions about alternative sources or potential HEV-1-like waterborne transmission routes for HEV-3c infections in humans. Hence, the distinct occurrence patterns of M1 (100% genotype 3) and M3 (100% genotype 1) motifs we report herein are consistent with the hypothesis that specific environmental and host-associated factors operate across ecological niches, potentially shaping capsid-level variation in relation to transmission routes and environmental persistence, beyond classical genotyping. Instead, these mutation motifs likely reflect constrained variability within a structurally sensitive region of the capsid where mutations may be tolerated while preserving overall virus particle integrity, especially for the M3 motif, as it does not disturb the assembly of HEV-like particles produced from recombinant ORF2 proteins (29). However, because these motifs occur at low frequencies, they are unlikely to represent a prerequisite for a particular transmission route, but may instead constitute one of several molecular factors contributing to environmental dissemination under specific ecological conditions. Future monitoring of variation within this ORF2 hinge-region HotSpot, particularly in clusters of waterborne HEV outbreaks, could help to determine whether the frequency of M1 and M3 motifs changes and/or co-varies with other viral genomic HotSpots in correlation with environmental persistence and water-linked transmission routes.

Second, the structural context of the residues 354, 357, and 364 provides a mechanistic framework for these observations. Located at the interface between the M and P domains of the ORF2 capsid protein (Fig. 3), this mutant-prone HotSpot (*HS*) appears to function as a flexible hinge that may influence the relative positioning and dynamics of the P domain (27). Concerning AF3 results, the sequence-based structure predictions can be interpreted as sampling a range of possible conformations (50). Consequently, the confidence metrics reflect the variability of these possible solutions, and analyzing them informs on the conformational landscape of ORF2. Interestingly, the M1 and M3 motifs clearly enhanced AF3 confidence metrics (Fig. 4), where these motifs are associated with reduced predicted alignment error of the P domain relative to the S and M domains, suggesting local conformational stabilization or altered domain organization. Such modifications may, in turn, affect the exposure or spatial arrangement of surface-exposed regions of HEV virions.

Third, in line with this structural interpretation, physicochemical analyses of model peptides revealed that substitutions within this hinge region are associated with increased hydrophobicity and detectable conformational changes (Fig. 5). These results support the idea that the M1 and M3 mutation motifs may induce alterations in surface properties of the ORF2 capsid protein. Moreover, because each motif is repeated 180 times across copies of ORF2 within the icosahedral *T =* 3 virion (27), these localized physicochemical changes are therefore propagated over the entire particle surface. Although the resulting impact on the net surface hydrophobicity of HEV particles remains to be experimentally determined, the symmetric architecture of viral capsids should amplify the cumulative effect of such local modifications, particularly through the association of newly hydrophobic M1 or M3 regions with pre-existing neighboring hydrophobic patches (55). In this context, these mutations should be considered collectively rather than individually as isolated changes. Consequently, given the importance of surface hydrophobicity with regard to immunogenicity of virus particles (56), as well as in governing particle stability, aggregation behavior, and interactions with surrounding media (57), such changes may influence the environmental behavior and persistence of HEV virions before they encounter susceptible host cells. During this extracellular phase, virions can experience non-specific interactions with surrounding biological and environmental matrices, including organic matter and mineral surfaces in environmental matrices (22, 23), as well as extracellular polymers and glycosaminoglycans such as heparan sulfate in host-associated environments (24). Consequently, even localized modifications of capsid physicochemical properties may alter the balance of these interactions and ultimately affect virus transport, persistence, and bioavailability for infection. From GRAVY analyses, we concluded that only the H354Y substitution, present both in the M1 and M3 motif, significantly modifies the hydrophobicity of the *HS* region of ORF2, whereas *in vitro* RP-HPLC analyses indicate that combined H354Y–G357S–V364I (M3 motif) further enhances this effect (Fig. 5d). This could be due to conformational changes as indicated by IR-ATR experiments (Fig. 5b) not captured by GRAVY scoring (58). Indeed, the spatial distribution of apolar residues may influence the functional hydrophobicity of proteins (59). This is illustrated through the H354Y substitution: the tyrosine (−1.3 HI), replacing the histidine (−3.2 HI), integrates into an already hydrophobic environment made from L353 (+3.8 HI) and F355 (+2.8 HI), then contributes to a continuous hydrophobic patch likely to exponentially increase the hydrophobicity of this region (55).

More broadly, because of the multifunctional nature of the ORF2 capsid protein, sequence variations may affect other aspects of HEV behavior during infection, including interactions with the host immune system, as recently shown by Joshi et al. (60).

Several limitations should nevertheless be considered in our conclusions. First, the low frequency (<3%) of the identified mutations limits the statistical power of comparative analyses and calls for cautious interpretation of observed trends. Second, structural predictions based on AF3 do not fully capture the dynamic nature of capsid assembly and should be interpreted as indicative rather than definitive. Third, physicochemical analyses were performed on isolated, model peptides, which do not entirely reproduce the intact virion. Further studies combining functional assays involving intact virions (e.g., infectivity assays and environmentally relevant persistence experiments) and full-particle modeling will therefore be necessary to determine whether the physicochemical differences identified here translate into measurable changes in biological behavior, environmental persistence, and transmission of HEV. Despite these limitations, this work highlights the value of integrating genetic, structural, and physicochemical approaches for investigating HEV circulation across environmental and host-associated matrices beyond genome-wide variation alone. By linking localized mutations to potential changes in capsid properties, our results provide a complementary perspective to genotype-based frameworks. In this context, the hinge region of the HEV ORF2 capsid protein emerges as a candidate site influencing virion surface properties and colloidal behavior, with M1 and M3 variation motifs potentially reflecting shifts in virion surface properties and environmental dissemination routes between HEV-3 and HEV-1.

### Conclusion

This study highlights how localized capsid features may shape the environmental behavior of HEV beyond genotype classification, especially when only short genomic sequences are available, which is frequent with environmental samples. The variations in the HEV ORF2 analyzed herein may influence the outer structure of virions, their surface physicochemical properties, and consequently their environmental persistence, dissemination, and interactions with hosts. These findings provide new opportunities for improving HEV surveillance through the identification of molecular signatures linking human, animal, and environmental reservoirs, and by integrating genetic, structural, and physicochemical analyses to better understand viral circulation within a One Health framework. More broadly, targeting key regions of viral capsid proteins provides a valuable approach for connecting molecular signatures to functional and ecological features of viruses. Such approaches may support future developments in both basic and applied research, including vaccine and antiviral drug design, as well as environmental monitoring and remediation strategies.

## MATERIALS AND METHODS

### European HEV sequence pooling and refinement for compiling the human-derived, animal-derived, and “water-linked” ORF2 sequence libraries

The European HEV sequences, accompanied by GenBank accession numbers and metadata, were extracted and pooled as follows: (i) European public databases were browsed, targeting sequences covering the M domain of the ORF2 gene and filtering the sequences as a function of their human or animal origin. This yielded the human-derived (*n* = 2,641) and the animal-derived (*n* = 1,184) ORF2 sequence libraries, both available in Tables S1 and S2. (ii) 702 additional sequences, accompanied by detailed clinical and epidemiological information, were compiled from 16 research articles mentioned in a recent meta-analysis reporting HEV in environmental water matrices (6) together with five further articles on the same topic (13, 30–49). For the purpose of the present study, these additional sequences (Table 1) were collectively designated as “water-linked” because they originated from studies describing HEV transmission pathways involving environmental waters, irrespective of whether the sequenced isolate itself originated from a human, animal, or environmental sample. As shown in Fig. 1, 28/702 sequences were insufficiently resolved to be aligned with the 674/702 other sequences and were therefore excluded. This initial filtering yielded 674 sequences from three sources: human (*n* = 274), animal (*n* = 307), and the environment (wastewaters and surface waters, *n* = 93), all involving water-linked transmission pathways between environmental, human, or animal compartments. A second refinement was performed to focus on the ORF2 gene, resulting in 422 ORF2-containing sequences. Finally, only the 336 complete or partial ORF2 sequences covering the M domain were selected from the 422 ORF2-containing sequences. This yielded the water-linked ORF2 sequence library (*n* = 336) available in Table S3. The selected HEV sequences came from nine European countries (France, Germany, Italy, Spain, the United Kingdom, Sweden, Slovenia, the Netherlands, and Portugal), to which data from Israel were added for comparison, as this country shares similarities with Western Europe, particularly in terms of medical care and wastewater treatment.

Moreover, seven sequences associated with French HEV strains were added from our local collection (DC-2016-2790 & 2018-A00117-48, Nancy, Clermont-Ferrand, Dijon) in accordance with international guidelines (61). The sequences come from patients and one wild boar. For the wild boar, a stool sample was collected in February 2023. The human samples, collected between the year 2017 and 2022, come from three patients from Clermont-Ferrand: (i) C500–02/2022; (ii) C954–11/2021; and (iii) C756–11/2021 and three patients from Dijon: (i) at two dates, *H5923–03/2019 and *H6011–05/2019; (ii) H4496–03/2017; and (iii) H6138–07/2019.

### Phylogenetic and molecular analyses of ORF2 sequences

Classical phylogeny was performed using the Molecular Evolutionary Genetic Analysis software version 12 (MEGA-12) (62) and re-reading of the related publications. The MEGA-12 data files of human-, animal-, and water-derived ORF2 libraries are available in Tables S1–S3). Phylogenetic and molecular analyses were performed on nucleotide sequences using the Maximum Likelihood method implemented in MEGA-12 (62), with the Kimura two-parameter model (63) and 1,000 bootstrap replicates. The amino acid residues of the ORF2 capsid protein were investigated to identify potential variable residues that could change HEV capsid surface properties, due to their functional role in HEV interactions with the cellular environment (*in vivo*) or with suspended particulate matter from the external aqueous environment (*ex vivo*). HEV sequences were originally typed by the corresponding article authors at the time of submission to GenBank, while HEV ORF2 sequences harboring M1 or M3 mutation motifs were re-examined with the online genotyping tool provided by the National Institute for Public Health and the Environment of Netherlands (RIVM, https://mpf.rivm.nl/mpf/typingtool/hev/) recently updated according to Smith et al. (3).

### AlphaFold structure predictions of the HEV ORF2 capsid protein

All the protein structure predictions were performed via the open-access AlphaFold-3 (AF3) web server (50). AF3 was used for modeling the mutated forms of the ORF2 capsid protein detected in the present study, based on the typical sequence published earlier (pdb_00002ztn; residues 129-606) (26). The raw AF3 data set was downloaded (available from the corresponding author upon request) and extracted with homemade Python scripts for analysis, while molecular models were visualized and reconstructed using the UCSF ChimeraX 1.10.1 software (64). Each fulfilled AF3 job came with a set of three confidence metrics used for assessing the veracity of the predicted protein structure: (i) first, the predicted Local Distance Difference Test (pLDDT) that considers distances of each residue to the proteinaceous backbone. The pLDDT per-residue score uses a 0–100 scale, where higher values indicate higher confidence. However, a score below 50 is already considered bad, meaning the position of the residue with respect to the proteinaceous backbone is probably wrong; (ii) the second metric is the PAE, assessing the deviation in the position of each residue with respect to all others in the predicted structure. The PAE score, expressed in angstrom units (Å), is consequently a paired metric between two residues where lower values indicate higher confidence. Plotting the PAE matrix as 3D heatmaps helps in visualizing the alignment errors in all pairs of residues in the predicted protein structure, while classic 2D plots consider the alignment error of one specific residue to all others; (iii) the predicted Template Modeling (pTM) score measures the overall precision of predictions. The pTM scores vary between 0 and 1, where a score above 0.5 means the overall predicted protein fold might be similar to the true structure.

### Model peptides

The impact of amino acid changes observed was explored for HEV surface structure and hydrophobicity on model peptides derived from the mutated region of the HEV capsid M domain. The model peptides LHFTGTNGVGEVG (N), LYFTGTNGVGEVG (M1), and LYFTSTNGVGEIG (M3) were synthesized at ProteoGenix SAS (Schiltigheim, France) with a purity grade >95% and shipped as lyophilized powder. A stock solution of each peptide (2 mg/mL) was prepared in 10 mM phosphate-buffered saline, pH 7, and stored at 4°C in the dark.

### IR-ATR of model peptides

IR-ATR was used for detecting structural changes in model peptides. Spectra were recorded on a Bruker Vertex70v spectrometer equipped with a KBr beam splitter and an MCT detector cooled with liquid N_2_. Measurements were performed at 22°C in an air-conditioned room. Spectra recording and data processing were performed using the Bruker OPUS 7.5 software. The resolution of the single-beam spectra was 4 cm^−1^. Two hundred scans were collected per spectrum, corresponding to a 2-min accumulation time. A nine-reflection diamond ATR accessory (DurasamplIR, SensIR Technologies, incidence angle: 45°) was used for acquiring spectra, without ATR correction. IR-ATR spectra are shown with an absorbance scale corresponding to Log(*R*_reference_/*R*_sample_), where *R* is the internal reflectance of the device. Fifty microliters of the peptide solution (1 mg/mL in 10 mM phosphate-buffered saline, pH 7, supplemented with 10 mM Brij L4) was put on the ATR crystal. The spectral background was removed by recording the spectrum of 10 mM phosphate-buffered saline supplemented with 10 mM Brij L4. Water vapor subtraction was performed when necessary. IR-ATR was used in aqueous solution, with a peptide solution that mimics partial solvent accessibility of the HS region in HEV virus particles (26, 27).

### Hydropathy indexes and RP-HPLC of model peptides

Theoretical hydropathy indexes (52) and RP-HPLC were used to compare the hydrophobicity of model peptides. RP-HPLC experiments were performed on a Thermo Fisher Scientific UltiMate 3000 HPLC System with UV detection at 220 nm wavelength (Thermo Fisher Scientific, Les Ulis, France) equipped with a Nucleosil 100-5 C18, 5 µm, 125 × 4 mm EC column (Macherey-Nagel, Hoerdt, France) and using a mobile phase mixture composed of HPLC-grade water and acetonitrile supplemented with 0.2% volume ratio of 85% aqueous phosphoric acid. Data acquisition and treatment were done with the Chromeleon software supplied with the HPLC system. The elution of each peptide or benzophenone sample (20 µL injection loop) was done at a 1 mL/min flow rate in gradient mode, starting with a water/acetonitrile 90/10 volume ratio and gradually switching to 50/50 over 30 min. The starting mobile phase was used for diluting peptide stock solutions from 2 to 0.05 mg/mL prior to injection. A benzophenone stock solution (2 mg/mL) was also prepared in the starting mobile phase and diluted to 0.05 mg/mL with the same solvent prior to analysis. All analyses were performed four times to ensure accuracy. Retention factors (*k*′) were calculated from each analysis as described elsewhere (65). The statistical comparisons (*P*-value) of Bzp-normalized *k*′ values were performed with GraphPad InStat v3.06 software (GraphPad Prism Software, San Diego, USA), using a two-way ANOVA test followed by Student’s post-test. A *P*-value< 0.001 (***) corresponds to a highly significant difference.

## Data Availability

Data generated or analyzed during this study are included in the article and its supplemental material. Other data and Python scripts are available from the corresponding author upon request.

## SUPPLEMENTAL MATERIAL

Table S1 aem.01445-26-s0001.xlsx Human-derived ORF2 library.

Table S2 aem.01445-26-s0002.xlsx Animal-derived ORF2 library.

Table S3 aem.01445-26-s0003.xlsx Water-linked ORF2 library.

Table S4 aem.01445-26-s0004.xlsx M1- and M3-harboring ORF2 sequences.

ASM does not own the copyrights to Supplemental Material that may be linked to, or accessed through, an article. The authors have granted ASM a non-exclusive, world-wide license to publish the Supplemental Material files. Please contact the corresponding author directly for reuse.

## References

[1] Songtanin B, Molehin AJ, Brittan K, Manatsathit W, Nugent K. 2023. Hepatitis E virus infections: epidemiology, genetic diversity, and clinical considerations. Viruses 15:1389. doi:10.3390/v15061389 PMC10304420 37376687

[2] Li P, Liu J, Li Y, Su J, Ma Z, Bramer WM, Cao W, de Man RA, Peppelenbosch MP, Pan Q. 2020. The global epidemiology of hepatitis E virus infection: a systematic review and meta-analysis. Liver Int 40:1516–1528. doi:10.1111/liv.14468 PMC7384095 32281721

[3] Smith DB, Simmonds P, Izopet J, Oliveira-Filho EF, Ulrich RG, Johne R, Koenig M, Jameel S, Harrison TJ, Meng X-J, Okamoto H, Van der Poel WHM, Purdy MA. 2016. Proposed reference sequences for hepatitis E virus subtypes. J Gen Virol 97:537–542. doi:10.1099/jgv.0.000393 PMC5588893 26743685

[4] Lu L, Li C, Hagedorn CH. 2006. Phylogenetic analysis of global hepatitis E virus sequences: genetic diversity, subtypes and zoonosis. Rev Med Virol 16:5–36. doi:10.1002/rmv.482 16175650

[5] Kamar N, Bendall R, Legrand-Abravanel F, Xia N-S, Ijaz S, Izopet J, Dalton HR. 2012. Hepatitis E. The Lancet 379:2477–2488. doi:10.1016/S0140-6736(11)61849-7 22549046

[6] Takuissu GR, Kenmoe S, Ndip L, Ebogo-Belobo JT, Kengne-Ndé C, Mbaga DS, Bowo-Ngandji A, Oyono MG, Kenfack-Momo R, Tchatchouang S, Kenfack-Zanguim J, Lontuo Fogang R, Zeuko’o Menkem E, Kame-Ngasse GI, Magoudjou-Pekam JN, Nkie Esemu S, Veneri C, Mancini P, Bonanno Ferraro G, Iaconelli M, Suffredini E, La Rosa G. 2022. Hepatitis E virus in water environments: a systematic review and meta-analysis. Food Environ Virol 14:223–235. doi:10.1007/s12560-022-09530-3 PMC9458591 36036329

[7] Brayne AB, Dearlove BL, Lester JS, Kosakovsky Pond SL, Frost SDW. 2017. Genotype-specific evolution of hepatitis E virus. J Virol 91:e02241-16. doi:10.1128/JVI.02241-16 PMC5391457 28202767

[8] Galle PR, Forner A, Llovet JM, Mazzaferro V, Piscaglia F, Raoul J-L, Schirmacher P, Vilgrain V. 2018. EASL clinical practice guidelines: management of hepatocellular carcinoma. J Hepatol 69:182–236. doi:10.1016/j.jhep.2018.03.019 29628281

[9] Mansuy JM, Gallian P, Dimeglio C, Saune K, Arnaud C, Pelletier B, Morel P, Legrand D, Tiberghien P, Izopet J. 2016. A nationwide survey of hepatitis E viral infection in French blood donors. Hepatology 63:1145–1154. doi:10.1002/hep.28436 27008201

[10] Pillonel J, Maugard C, Sommen C, Figoni J, Pierre C, LeCam S, Richard P, Morel P, Gallian P, Laperche S. 2022. Risk of a blood donation contaminated with hepatitis E virus entering the blood supply before the implementation of universal RNA screening in France. Vox Sang 117:1411–1414. doi:10.1111/vox.13375 36394899

[11] Capai L, Hozé N, Chiaroni J, Gross S, Djoudi R, Charrel R, Izopet J, Bosseur F, Priet S, Cauchemez S, de Lamballerie X, Falchi A, Gallian P. 2020. Seroprevalence of hepatitis E virus among blood donors on Corsica, France, 2017. Euro Surveill 25:1900336. doi:10.2807/1560-7917.ES.2020.25.5.1900336 PMC7014670 32046820

[12] Fenaux H, Chassaing M, Berger S, Jeulin H, Gentilhomme A, Bensenane M, Bronowicki JP, Gantzer C, Bertrand I, Schvoerer E. 2018. Molecular features of hepatitis E virus circulation in environmental and human samples. J Clin Virol 103:63–70. doi:10.1016/j.jcv.2018.04.003 29656087

[13] Hartard C, Fenaux H, Gentilhomme A, Murray JM, Akand E, Laugel E, Berger S, Maul A, de Rougemont A, Jeulin H, Remen T, Bensenane M, Bronowicki J-P, Gantzer C, Bertrand I, Schvoerer E. 2021. Variability in molecular characteristics of hepatitis E virus quasispecies could modify viral surface properties and transmission. J Viral Hepat 28:1078–1090. doi:10.1111/jvh.13513 33877740

[14] Saint-Jacques P, Tissot-Dupont H, Colson P. 2016. Autochthonous infection with hepatitis E virus related to subtype 3a, France: a case report. Ann Hepatol 15:438–441. doi:10.5604/16652681.1198823 27049499

[15] Khuroo MS, Khuroo MS, Khuroo NS. 2016. Hepatitis E: discovery, global impact, control and cure. World J Gastroenterol 22:7030–7045. doi:10.3748/wjg.v22.i31.7030 PMC4988308 27610014

[16] Primadharsini PP, Nagashima S, Okamoto H. 2019. Genetic variability and evolution of hepatitis E virus. Viruses 11:456. doi:10.3390/v11050456 PMC6563261 31109076

[17] Murray JM, Moenne-Loccoz R, Velay A, Habersetzer F, Doffoël M, Gut J-P, Fofana I, Zeisel MB, Stoll-Keller F, Baumert TF, Schvoerer E. 2013. Genotype 1 hepatitis C virus envelope features that determine antiviral response assessed through optimal covariance networks. PLoS One 8:e67254. doi:10.1371/journal.pone.0067254 PMC3688619 23840641

[18] Murray JM, Murray DD, Schvoerer E, Akand EH. 2024. SARS-CoV-2 delta and omicron community transmission networks as added value to contact tracing. J Infect 88:173–179. doi:10.1016/j.jinf.2024.01.004 38242366

[19] Zhang M, Altan-Bonnet N, Shen Y, Shuai D. 2022. Waterborne human pathogenic viruses in complex microbial communities: environmental implication on virus infectivity, persistence, and disinfection. Environ Sci Technol 56:5381–5389. doi:10.1021/acs.est.2c00233 PMC9073700 35434991

[20] Wang B, Meng X-J. 2021. Structural and molecular biology of hepatitis E virus. Comput Struct Biotechnol J 19:1907–1916. doi:10.1016/j.csbj.2021.03.038 PMC8079827 33995894

[21] van Tong H, Hoan NX, Wang B, Wedemeyer H, Bock C-T, Velavan TP. 2016. Hepatitis E virus mutations: functional and clinical relevance. EBioMedicine 11:31–42. doi:10.1016/j.ebiom.2016.07.039 PMC5049923 27528267

[22] Schijven JF, Hassanizadeh SM. 2000. Removal of viruses by soil passage: overview of modeling, processes, and parameters. Crit Rev Environ Sci Technol 30:49–127. doi:10.1080/10643380091184174

[23] Heffron J, Mayer BK. 2016. Emerging investigators series: virus mitigation by coagulation: recent discoveries and future directions. Environ Sci: Water Res Technol 2:443–459. doi:10.1039/C6EW00060F

[24] Cagno V, Tseligka ED, Jones ST, Tapparel C. 2019. Heparan sulfate proteoglycans and viral attachment: true receptors or adaptation bias? Viruses 11:596. doi:10.3390/v11070596 PMC6669472 31266258

[25] Cancela F, Noceti O, Arbiza J, Mirazo S. 2022. Structural aspects of hepatitis E virus. Arch Virol 167:2457–2481. doi:10.1007/s00705-022-05575-8 PMC9469829 36098802

[26] Yamashita T, Mori Y, Miyazaki N, Cheng RH, Yoshimura M, Unno H, Shima R, Moriishi K, Tsukihara T, Li TC, Takeda N, Miyamura T, Matsuura Y. 2009. Biological and immunological characteristics of hepatitis E virus-like particles based on the crystal structure. Proc Natl Acad Sci USA 106:12986–12991. doi:10.1073/pnas.0903699106 PMC2722322 19620712

[27] Xing L, Li TC, Mayazaki N, Simon MN, Wall JS, Moore M, Wang CY, Takeda N, Wakita T, Miyamura T, Cheng RH. 2010. Structure of hepatitis E virion-sized particle reveals an RNA-dependent viral assembly pathway. J Biol Chem 285:33175–33183. doi:10.1074/jbc.M110.106336 PMC2963395 20720013

[28] Zhang S, Qu C, Wang Y, Wang W, Ma Z, Peppelenbosch MP, Pan Q. 2018. Conservation and variation of the hepatitis E virus ORF2 capsid protein. Gene 675:157–164. doi:10.1016/j.gene.2018.06.108 30180962

[29] Kobayashi T, Takahashi M, Ohta S, Hoshino Y, Yamada K, Jirintai S, Primadharsini PP, Nagashima S, Murata K, Okamoto H. 2024. Production and characterization of self-assembled virus-like particles comprising capsid proteins from genotypes 3 and 4 hepatitis E virus (HEV) and rabbit HEV expressed in *Escherichia coli*. Viruses 16:1400. doi:10.3390/v16091400 PMC11437457 39339876

[30] Lhomme S, Magne S, Perelle S, Vaissière E, Abravanel F, Trelon L, Hennechart-Collette C, Fraisse A, Martin-Latil S, Izopet J, Figoni J, Spaccaferri G. 2023. Clustered cases of waterborne hepatitis E virus infection, France. Viruses 15:1149. doi:10.3390/v15051149 PMC10224386 37243235

[31] Bisseux M, Colombet J, Mirand A, Roque-Afonso A-M, Abravanel F, Izopet J, Archimbaud C, Peigue-Lafeuille H, Debroas D, Bailly J-L, Henquell C. 2018. Monitoring human enteric viruses in wastewater and relevance to infections encountered in the clinical setting: a one-year experiment in central France, 2014 to 2015. Euro Surveill 23:17-00237. doi:10.2807/1560-7917.ES.2018.23.7.17-00237 PMC5824128 29471623

[32] Beyer S, Szewzyk R, Gnirss R, Johne R, Selinka H-C. 2020. Detection and characterization of hepatitis E virus genotype 3 in wastewater and urban surface waters in Germany. Food Environ Virol 12:137–147. doi:10.1007/s12560-020-09424-2 PMC7225198 32172512

[33] De Sabato L, Di Bartolo I, Lapa D, Capobianchi MR, Garbuglia AR. 2020. Molecular characterization of HEV genotype 3 in Italy at human/animal interface. Front Microbiol 11:137. doi:10.3389/fmicb.2020.00137 PMC7014918 32117156

[34] Zecchin B, Schivo A, Milani A, Fusaro A, Zamperin G, Bellinati L, Ceglie L, Natale A, Bonfanti L, Cunial G, Obber F, Di Bartolo I, Citterio C, Monne I. 2019. Identification of a zoonotic genotype 3 hepatitis E subtype in wildlife in north-eastern Italy. Infection, Genetics and Evolution 71:16–20. doi:10.1016/j.meegid.2019.03.005 30876888

[35] Veneri C, Brandtner D, Mancini P, Bonanno Ferraro G, Iaconelli M, Del Giudice C, Ciccaglione AR, Bruni R, Equestre M, Marcantonio C, Suffredini E, La Rosa G. 2024. Detection and full genomic sequencing of rare hepatitis E virus genotype 4d in Italian wastewater, undetected by clinical surveillance. Sci Total Environ 913:169698. doi:10.1016/j.scitotenv.2023.169698 38160838

[36] Iaconelli M, Purpari G, Della Libera S, Petricca S, Guercio A, Ciccaglione AR, Bruni R, Taffon S, Equestre M, Fratini M, Muscillo M, La Rosa G. 2015. Hepatitis A and E viruses in wastewaters, in river waters, and in bivalve molluscs in Italy. Food Environ Virol 7:316–324. doi:10.1007/s12560-015-9207-3 26115693

[37] Iaconelli M, Bonanno Ferraro G, Mancini P, Suffredini E, Veneri C, Ciccaglione AR, Bruni R, Della Libera S, Bignami F, Brambilla M, De Medici D, Brandtner D, Schembri P, D’Amato S, La Rosa G. 2020. Nine-year nationwide environmental surveillance of hepatitis E virus in urban wastewaters in Italy (2011–2019). Int J Environ Res Public Health 17:2059. doi:10.3390/ijerph17062059 PMC7143501 32244915

[38] Ram D, Manor Y, Gozlan Y, Schwartz E, Ben-Ari Z, Mendelson E, Mor O. 2016. Hepatitis E virus genotype 3 in sewage and genotype 1 in acute hepatitis cases, Israel. Am J Trop Med Hyg 95:216–220. doi:10.4269/ajtmh.15-0925 PMC4944693 27246446

[39] Idolo A, Serio F, Lugoli F, Grassi T, Bagordo F, Guido M, Privitera G, Lobreglio G, De Donno A. 2013. Identification of HEV in symptom-free migrants and environmental samples in Italy. J Viral Hepat 20:438–443. doi:10.1111/jvh.12038 23647961

[40] La Rosa G, Pourshaban M, Iaconelli M, Vennarucci VS, Muscillo M. 2010. Molecular detection of hepatitis E virus in sewage samples. Appl Environ Microbiol 76:5870–5873. doi:10.1128/AEM.00336-10 PMC2935059 20601505

[41] Rutjes SA, Lodder WJ, Lodder-Verschoor F, van den Berg H, Vennema H, Duizer E, Koopmans M, de Roda Husman AM. 2009. Sources of hepatitis E virus genotype 3 in The Netherlands. Emerg Infect Dis 15:381–387. doi:10.3201/eid1503.071472 PMC2681103 19239749

[42] Matos A, Mesquita JR, Gonçalves D, Abreu-Silva J, Luxo C, Nascimento MS. 2018. First detection and molecular characterization of hepatitis E virus in water from wastewater treatment plants in Portugal. Ann Agric Environ Med 25:364–367. doi:10.26444/aaem/90497 29936796

[43] Steyer A, Naglič T, Močilnik T, Poljšak-Prijatelj M, Poljak M. 2011. Hepatitis E virus in domestic pigs and surface waters in slovenia: prevalence and molecular characterization of a novel genotype 3 lineage. Infect Genet Evol 11:1732–1737. doi:10.1016/j.meegid.2011.07.007 21802527

[44] Clemente-Casares P, Pina S, Buti M, Jardi R, MartIn M, Bofill-Mas S, Girones R. 2003. Hepatitis E virus epidemiology in industrialized countries. Emerg Infect Dis 9:448–454. doi:10.3201/eid0904.020351 PMC2957966 12702225

[45] Pina S, Buti M, Cotrina M, Piella J, Girones R. 2000. HEV identified in serum from humans with acute hepatitis and in sewage of animal origin in Spain. J Hepatol 33:826–833. doi:10.1016/s0168-8278(00)80316-5 11097493

[46] Pina S, Jofre J, Emerson SU, Purcell RH, Girones R. 1998. Characterization of a strain of infectious hepatitis E virus isolated from sewage in an area where hepatitis E is not endemic. Appl Environ Microbiol 64:4485–4488. doi:10.1128/AEM.64.11.4485-4488.1998 PMC106673 9797311

[47] Randazzo W, Vásquez-García A, Bracho MA, Alcaraz MJ, Aznar R, Sánchez G. 2018. Hepatitis E virus in lettuce and water samples: a method-comparison study. Int J Food Microbiol 277:34–40. doi:10.1016/j.ijfoodmicro.2018.04.008 29680694

[48] Wang H, Neyvaldt J, Enache L, Sikora P, Mattsson A, Johansson A, Lindh M, Bergstedt O, Norder H. 2020. Variations among viruses in influent water and effluent water at a wastewater plant over one year as assessed by quantitative PCR and metagenomics. Appl Environ Microbiol 86:e02073-20. doi:10.1128/AEM.02073-20 PMC7688244 33036988

[49] Smith DB, Paddy JO, Simmonds P. 2016. The use of human sewage screening for community surveillance of hepatitis E virus in the UK. J Med Virol 88:915–918. doi:10.1002/jmv.24403 PMC4832372 26461450

[50] Abramson J, Adler J, Dunger J, Evans R, Green T, Pritzel A, Ronneberger O, Willmore L, Ballard AJ, Bambrick J, et al.. 2024. Accurate structure prediction of biomolecular interactions with AlphaFold 3. Nature 630:493–500. doi:10.1038/s41586-024-07487-w PMC11168924 38718835

[51] Kong J, Yu S. 2007. Fourier transform infrared spectroscopic analysis of protein secondary structures. Acta Biochim Biophys Sin (Shanghai) 39:549–559. doi:10.1111/j.1745-7270.2007.00320.x 17687489

[52] Kyte J, Doolittle RF. 1982. A simple method for displaying the hydropathic character of a protein. J Mol Biol 157:105–132. doi:10.1016/0022-2836(82)90515-0 7108955

[53] Mant CT, Kovacs JM, Kim H-M, Pollock DD, Hodges RS. 2009. Intrinsic amino acid side-chain hydrophilicity/hydrophobicity coefficients determined by reversed-phase high-performance liquid chromatography of model peptides: comparison with other hydrophilicity/hydrophobicity scales. Biopolymers 92:573–595. doi:10.1002/bip.21316 PMC2792893 19795449

[54] Wang H, Churqui MP, Taslimi S, Tunovic T, Andius LD, Lagging M, Nyström K. 2025. Distinct distribution of HEV-3 subtypes across humans, animals, and environmental waters in Sweden. Emerg Microbes Infect 14:2488188. doi:10.1080/22221751.2025.2488188 PMC12001855 40166982

[55] Jacak R, Leaver-Fay A, Kuhlman B. 2012. Computational protein design with explicit consideration of surface hydrophobic patches. Proteins 80:825–838. doi:10.1002/prot.23241 PMC3277657 22223219

[56] Berthier L, Brass O, Deleage G, Terreux R. 2020. Construction of atomic models of full hepatitis B vaccine particles at different stages of maturation. J Mol Graph Model 98:107610. doi:10.1016/j.jmgm.2020.107610 32302938

[57] Armanious A, Aeppli M, Jacak R, Refardt D, Sigstam T, Kohn T, Sander M. 2016. Viruses at solid-water interfaces: a systematic assessment of interactions driving adsorption. Environ Sci Technol 50:732–743. doi:10.1021/acs.est.5b04644 26636722

[58] Sautrey G. 2025. An update on theoretical and metrological aspects of the surface hydrophobicity of virus and virus-like particles. Adv Biol (Weinh) 9:e2400221. doi:10.1002/adbi.202400221 39435562

[59] Rego NB, Ferguson AL, Patel AJ. 2022. Learning the relationship between nanoscale chemical patterning and hydrophobicity. Proc Natl Acad Sci USA 119:e2200018119. doi:10.1073/pnas.2200018119 PMC9860318 36409904

[60] Joshi G, Décembre E, Brocard J, Montpellier C, Ferrié M, Allatif O, Mehnert A-K, Pons J, Galiana D, Dao Thi VL, Jouvenet N, Cocquerel L, Dreux M. 2025. Plasmacytoid dendritic cell sensing of hepatitis E virus is shaped by both viral and host factors. Life Sci Alliance 8:e202503256. doi:10.26508/lsa.202503256 PMC11966012 40175091

[61] WMA declaration of Helsinki. Available from: https://www.wma.net/policies-post/wma-declaration-of-helsinki. Retrieved 21 Jan 2026.

[62] Kumar S, Stecher G, Suleski M, Sanderford M, Sharma S, Tamura K. 2024. MEGA12: molecular evolutionary genetic analysis version 12 for adaptive and green computing. Mol Biol Evol 41:msae263. doi:10.1093/molbev/msae263 PMC11683415 39708372

[63] Kimura M. 1980. A simple method for estimating evolutionary rates of base substitutions through comparative studies of nucleotide sequences. J Mol Evol 16:111–120. doi:10.1007/BF01731581 7463489

[64] Pettersen EF, Goddard TD, Huang CC, Meng EC, Couch GS, Croll TI, Morris JH, Ferrin TE. 2021. UCSF ChimeraX: structure visualization for researchers, educators, and developers. Protein Sci 30:70–82. doi:10.1002/pro.3943 PMC7737788 32881101

[65] Idei M, Seprődi J, Győrffy E, Hollósy F, Vadász Z, Mészáros G, Teplán I, Kéri G. 1998. Micellar electrokinetic chromatography of highly hydrophobic peptides. Anal Chim Acta 372:273–279. doi:10.1016/S0003-2670(98)00338-9

